# Deprescribing Strategies for Opioids and Benzodiazepines with Emphasis on Concurrent Use: A Scoping Review

**DOI:** 10.3390/jcm12051788

**Published:** 2023-02-23

**Authors:** Yanning Wang, Debbie L. Wilson, Deanna Fernandes, Lauren E. Adkins, Ashley Bantad, Clint Copacia, Nilay Dharma, Pei-Lin Huang, Amanda Joseph, Tae Woo Park, Jeffrey Budd, Senthil Meenrajan, Frank A. Orlando, John Pennington, Siegfried Schmidt, Ronald Shorr, Constance R. Uphold, Wei-Hsuan Lo-Ciganic

**Affiliations:** 1 Department of Pharmaceutical Outcomes and Policy, College of Pharmacy, University of Florida, Gainesville, FL 32610, USA; 2 Department of Health Outcome and Biomedical Informatics, College of Medicine, University of Florida, Gainesville, FL 32610, USA; 3 North Florida/South Georgia Veterans Health System Geriatric Research Education and Clinical Center, Gainesville, FL 32601, USA; 4 Health Science Center Libraries, University of Florida, Gainesville, FL 32610, USA; 5 College of Pharmacy, University of Florida, Gainesville, FL 32610, USA; 6 Department of Psychiatry, University of Pittsburgh, Pittsburgh, PA 15213, USA; 7 Department of Medicine, College of Medicine, University of Florida, Gainesville, FL 32611, USA; 8 Department of Community Heath and Family Medicine, College of Medicine, University of Florida, Gainesville, FL 32608, USA; 9 Department of Epidemiology, College of Public Health and Health Professions & College of Medicine, University of Florida, Gainesville, FL 32610, USA; 10 Department of Physiology and Aging, College of Medicine, University of Florida, Gainesville, FL 32610, USA; 11 Center for Drug Evaluation and Safety (CoDES), College of Pharmacy, University of Florida, Gainesville, FL 32610, USA

**Keywords:** opioid, benzodiazepine, concurrent use, deprescribing, tapering

## Abstract

While the Food and Drug Administration’s black-box warnings caution against concurrent opioid and benzodiazepine (OPI–BZD) use, there is little guidance on how to deprescribe these medications. This scoping review analyzes the available opioid and/or benzodiazepine deprescribing strategies from the PubMed, EMBASE, Web of Science, Scopus, and Cochrane Library databases (01/1995–08/2020) and the gray literature. We identified 39 original research studies (opioids: n = 5, benzodiazepines: n = 31, concurrent use: n = 3) and 26 guidelines (opioids: n = 16, benzodiazepines: n = 11, concurrent use: n = 0). Among the three studies deprescribing concurrent use (success rates of 21–100%), two evaluated a 3-week rehabilitation program, and one assessed a 24-week primary care intervention for veterans. Initial opioid dose deprescribing rates ranged from (1) 10–20%/weekday followed by 2.5–10%/weekday over three weeks to (2) 10–25%/1–4 weeks. Initial benzodiazepine dose deprescribing rates ranged from (1) patient-specific reductions over three weeks to (2) 50% dose reduction for 2–4 weeks, followed by 2–8 weeks of dose maintenance and then a 25% reduction biweekly. Among the 26 guidelines identified, 22 highlighted the risks of co-prescribing OPI–BZD, and 4 provided conflicting recommendations on the OPI–BZD deprescribing sequence. Thirty-five states’ websites provided resources for opioid deprescription and three states’ websites had benzodiazepine deprescribing recommendations. Further studies are needed to better guide OPI–BZD deprescription.

## 1. Introduction

Opioid overdose remains a pervasive public health problem in the United States (US), with over 80,000 estimated opioid overdose deaths in 2021 [1]. Approximately 18% of opioid overdose deaths involve prescription opioids [2], and 31% to 61% involve benzodiazepines [3,4,5]. Additionally, about 38% of benzodiazepine overdose deaths involve prescription opioids [6]. Prior studies found that concurrent opioid and benzodiazepine use was associated with a 1.5- to 10-fold elevated overdose risk compared to opioid use alone [4,7,8,9,10,11]. 

Even with clinical guidelines and US Food and Drug Administration (FDA) black-box warnings cautioning against concurrent opioid and benzodiazepine use [12,13,14], many individuals use them concurrently (e.g., 25% of long-term opioid users concomitantly use benzodiazepines) [15]. Clinicians face challenges in avoiding such co-prescription in certain patients (e.g., chronic pain with anxiety or sleep disorders), despite known synergistic respiratory-depression effects and increased adverse health outcome risks (e.g., falls) [12,13,14]. Furthermore, opioid-related policies and restrictions may result in unintended consequences (e.g., abrupt discontinuation). In 2019, the FDA issued a drug safety communication calling for gradual, individualized opioid tapering due to potential harms (e.g., overdose and mental health crisis) from abrupt opioid dose reduction that may lead some patients to seek illicit sources [16]. In 2020, the FDA also updated the boxed warning on all benzodiazepines, cautioning against abrupt discontinuation due to withdrawal reactions such as chest pain, rapid heart rate, shaking, and agoraphobia [17]. 

Deprescribing is the systematic reduction in dose or discontinuation of a medication [18,19]. Systematic reviews and guidelines have widely varying recommendations on tapering, deprescribing, or discontinuing opioids and benzodiazepines separately. Little is known about strategies and evidence available for deprescribing concurrent opioid and benzodiazepine use. We conducted a scoping review of the published, peer-reviewed literature and the publicly available, web-based gray literature (e.g., governmental guidelines, clinical protocols) to collate the available evidence and strategies on deprescribing opioids and/or benzodiazepines to better guide clinical care and target interventions toward concurrent users.

## 2. Materials and Methods

### 2.1. Data Sources and Search Strategies

This scoping review complied with the Preferred Reporting Items for Systematic reviews and Meta-Analyses extension for Scoping Reviews (PRISMA-ScR) reporting guidelines [20]. The study protocol was registered with the Open Science Framework (10.17605/OSF.IO/ZUMND). 

A health sciences librarian (L.E.A) performed the original search, in August 2020, of the PubMed, EMBASE, Web of Science, Scopus, and Cochrane Library databases for studies published from January 1995 to August 2020. The search strategy combined database-specific, controlled vocabulary and used truncation and phrase searching in titles and abstracts for articles on deprescribing opioids and benzodiazepines in concurrent and nonconcurrent use. We restricted the search results to the English language. The PubMed search strategy is available in Supplementary Table S1. Additionally, we used relevant keywords (e.g., opioid deprescribing or tapering) to search publicly available, web-based gray literature including deprescribing guidelines or protocols published from (1) the Canadian Deprescribing Network [21] and the US Deprescribing Research Network [22], (2) the US federal government agencies (e.g., Centers for Disease Control and Prevention) and the state health departments (Table S2), and (3) leading health systems and health insurance companies based on net patient revenue or number of members identified by Statista [23] and Definitive Healthcare [24]. Finally, we manually screened reference lists from eligible identified sources.

### 2.2. Study Selection, Data Extraction, and Data Synthesis

We restricted our search to clinical trials or observational studies reporting details of deprescribing strategies. Such strategies included deprescribing speed and dose reduction specifications in ambulatory adults aged ≥ 18 years using opioids or benzodiazepines. We excluded studies focusing on the deprescription of buprenorphine or methadone use for opioid use disorder. 

After a comprehensive literature search and removal of duplicates, at least two investigators (D.L.W., A.B., C.C., N.D., P.-L.H., and A.J.) independently screened each source and extracted data. We screened the articles’ titles, abstracts, and full text using Covidence (Melbourne, Australia) and extracted information of interest using a standardized Microsoft Excel spreadsheet. For original studies, we extracted the author, publication year, country, deprescribing focus (opioid, benzodiazepine, or both), reasons for deprescribing, deprescribing protocols, the successful proportion deprescribed, discontinuation duration, and other deprescribing interventions used in the study (e.g., medication and cognitive behavioral therapy). Given that several published systematic and scoping reviews summarize deprescribing strategies for opioids or benzodiazepines alone [25,26,27,28,29,30], we focused on reporting the studies that deprescribed concurrent opioid and benzodiazepine use. For the guidelines/protocols, we extracted deprescribing details, including criteria for considering deprescription, alternative medication therapies, nondrug approaches, and management methods of withdrawal symptoms. We also summarized how states and leading insurance companies referenced or adapted the existing national guidelines. When there were multiple versions of a guideline/protocol available from one entity, we included the information from the most recent version. Disagreements in data extracted between reviewers were resolved by a third investigator (D.L.W. or W.-H.L.-C.). 

## 3. Results

**Figure 1:**
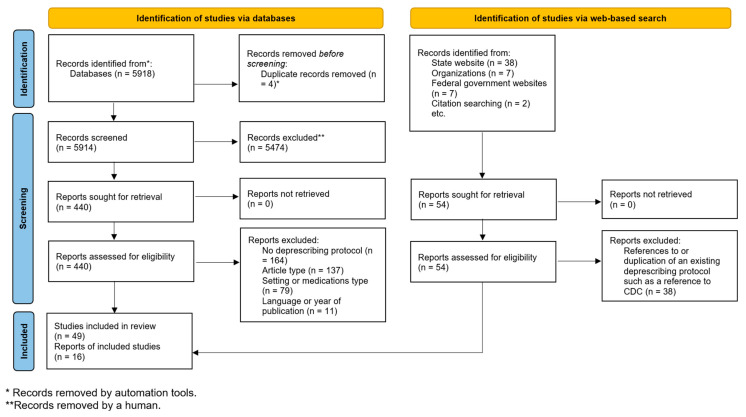
Flow diagram of included studies.

As shown in Figure 1, our peer-reviewed literature search identified 5914 articles after removing duplicates. Title and abstract screening excluded 5474 studies. We reviewed the full text of 440 articles and included 39 eligible original research articles and 10 practice guidelines. Our gray literature search identified 16 unique governmental or health systems’ deprescribing guidelines. 

### 3.1. Original Research Studies

Of the 39 studies, 14 (36%) were conducted in the US and 10 (26%) in Canada (Table S3). The publication years ranged from 1995 to 2019. Overall, 5 studies focused on opioid deprescription only, and 31 focused on benzodiazepine deprescription only. Below, we describe in detail three studies (Cunningham et al., 2016 [31]; Gilliam et al., 2018 [32]; Zaman et al., 2018 [33]) that used strategies to deprescribe both medications when used concomitantly (Table S4). 

Cunningham et al. (2016) [31] evaluated an intensive 3-week outpatient interdisciplinary rehabilitation program for discontinuing opioids in individuals with a primary diagnosis of fibromyalgia and chronic noncancer pain in a pain rehabilitation center in the US. Individuals in the opioid sample (n = 55) were on average 49 years old and mostly of Caucasian race (91.0%) and female (84.0%). Deprescribing recommendations varied depending on the patients’ morphine equivalent daily dose (MME/day). Patients with ≤100 MME/day were deprescribed off opioids over a mean of 10 days; those taking 100–200 MME/day were deprescribed over a mean of 14 days; and those taking >200 MME/day were deprescribed over a mean of 18 days. From day 1 to day 7 or 11 of the 3-week deprescribing period, participants generally reduced their total opioid dose by 10–20% each weekday (Monday–Friday). They reduced the dose by 2.5–10% each weekday during the remaining taper period. Of the 55 taking opioids, 51 (92.7%) discontinued the opioids during the 3-week program. With the goal of discontinuation of opioid and adjuvant medications for chronic nonmalignant pain, benzodiazepines were deprescribed by developing a deprescribing schedule individualized to each patient. If, at program discharge, the patient had started to reduce their benzodiazepine dose, a generic recommendation was used in the summary note that (1) indicated the initial dose and the current dose and (2) recommended that the primary care provider (PCP) continue the current dose for the next 2–4 weeks and then continue to taper the patient by approximately 25% every 2–4 weeks until completed. The proportion of persons concurrently taking benzodiazepines and opioids and the outcomes of benzodiazepine deprescription were not measured. 

Gilliam et al. (2018) [32] evaluated a 3-week interdisciplinary rehabilitation program intervention in noncancer pain patients in the same pain rehabilitation center where the Cunningham et al. (2016) study was conducted [31]. Individuals in the Gilliam et al. (2018) [32] sample who completed the program (285/344 [82.8%]) were on average 49 years old and mostly of Caucasian race (88.7%) and female (62.8%). Among 142 patients taking opioids at baseline who completed the program, 58 (40.8%) were also taking benzodiazepines at baseline. The study also included 43 individuals, who were taking benzodiazepines but not opioids (n = 143) at baseline, who completed the program. The opioid deprescribing schedules reduced doses by 10–20% each weekday from day 1 to day 7 or 11 of the 15-day taper and by 2.5–10% each weekday during the remainder. The benzodiazepine deprescribing protocol was the same as that in the Cunningham (2016) study. The article mentioned that benzodiazepine deprescription could be extended beyond the 3-week program because the risk for complications is greater for benzodiazepine tapers than for opioid tapers. Of the 165 patients taking opioids, 142 (86.1%) completed the deprescribing protocol and discontinued the opioids. Among 58 patients taking opioids and benzodiazepines concurrently at baseline, all (100%) discontinued opioids, and 20 (34.5%) discontinued both medications. Among 43 patients taking benzodiazepines but not taking opioids at baseline, 23 (53.4%) discontinued benzodiazepines.

Zaman et al. (2018) [33] evaluated an electronic intervention in a US Veteran cohort (61% White race and 92% male) receiving primary care in one US Veterans’ administration system and co-prescribed opioids (>90 days in the prior 120 days) and benzodiazepines (defined as having ≥1 overlapping day using both medication classes). Individuals in the sample (n = 145) were on average 62 years old and had a high incidence of mood (61%) and post-traumatic stress (56%) disorders. The intervention consisted of a review note in the patient’s electronic medical record (EMR), an email to the prescriber, and a clinician’s guide on deprescribing opioids and benzodiazepines. The EMR review note and email recommendations for deprescription asked clinicians to “consider benzodiazepine and/or opioid taper (to <100 MME/day or off entirely if still prescribed benzodiazepine)”. The opioid deprescribing schedule in the clinician’s guide recommended reducing the dose by 10–25% every 1 to 4 weeks for most patients and to rapidly reduce the dose every 1 to 7 days in medically dangerous situations. The benzodiazepine deprescribing schedule in the clinician’s guide recommended reducing the dose by 50% for 2 to 4 weeks, then maintaining the dose for 1 to 2 months, and then reducing it by 25% every 2 weeks. Conversion to a long-acting benzodiazepine was recommended as an alternative for patients who were not concurrently prescribed high-dose opioids. The proportion of individuals with ≥100 MME/day at baseline (39/145 [26.8%]) decreased by 30% at six months (26/139 [18.7%]). The mean MME/day decreased from 84.6 MME/day at baseline to 76.2 MME/day at 3-month follow up and to 65.6 MME/day at 6-month follow up, resulting in a 22% decrease over six months. The mean diazepam milligram equivalent daily dose (DME/day) decreased from 16.10 DME/day at baseline to 13.8 DME/day at 3-month follow up and to 13.4 DME/day at 6-month follow up, resulting in a 17% decrease over six months. The number of individuals with co-prescribed opioids and benzodiazepines decreased from baseline by 21% at 3-month follow up. At the 6-month measurement (n = 139), 46 (33.1%) patients were no longer co-prescribed opioids and benzodiazepines (14 [10.1%] discontinued opioids, 23 [16.5%] discontinued benzodiazepines, and 9 [6.5%] discontinued both). 

### 3.2. Guidelines

Among the 26 unique guidelines identified, 16 provided deprescribing schedules for opioids only, and 11 provided deprescribing schedules for benzodiazepines only, with the Oregon Pain Guidance [34] guideline mentioning both medication classes (Table 1). All 26 guidelines included criteria for when to consider deprescription and emphasized the importance of withdrawal symptom management, shared decision-making with patients, and nonpharmacological support. In all, 14 of the 16 (88%) opioid deprescribing guidelines and 9 of 11 benzodiazepine guidelines mentioned the risk of concurrent opioid and benzodiazepine use, but none of the 26 had specific deprescribing schedules for concurrent users. Two guidelines recommended that concomitant users deprescribe opioids first, followed by gradual benzodiazepine deprescription [35,36], while the Oregon Pain Guidance [34] guideline recommended the opposite and the Minnesota Department of Human Services website suggested that benzodiazepines may be deprescribed first for patients receiving concurrent high daily MME doses and intermittent benzodiazepines [37]. The Minnesota Department of Human Services website did not define high daily MME in concurrent benzodiazepine use.

**Figure 2:**
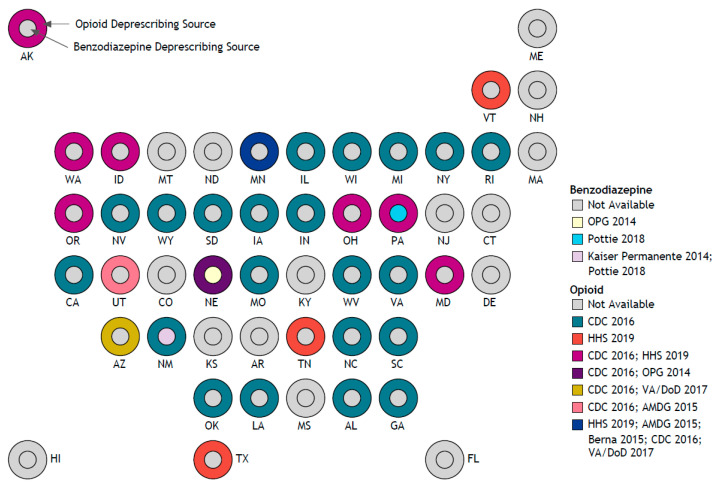
Deprescribing sources of opioid and benzodiazepine by state.

As shown in Figure 2, for opioid deprescription, 35 (70%) of the 50 US states provided resources on their state health department websites, with a majority referencing the CDC 2016 guideline [64] (n = 32) and the US Department of Health and Human Services (HHS) 2019 guideline [44] (n = 11), and 5 made additional recommendations [35,36,37,40,41]. For benzodiazepine deprescription, three states provide resources—Nebraska, New Mexico, and Pennsylvania—adapted from Oregon Pain Guidance [34], Kaiser Permanente [51], and Pottie et al. (2018) [54], respectively. None of the states’ health department websites provided a deprescribing schedule for concurrent opioid and benzodiazepine users.

## 4. Discussion

The findings from our scoping review underscore the importance of developing evidence-based guidance on deprescribing concurrent opioid and benzodiazepine use in clinical practice. Of the 39 original, peer-reviewed research articles, only 3 discussed strategies to deprescribe both medications when used concomitantly, and of the 26 unique practice guidelines for deprescribing opioids and/or benzodiazepines, only 4 mentioned deprescribing concurrent opioid and benzodiazepine use. The deprescribing recommendations of the three original, peer-reviewed articles were widely divergent on how fast to reduce the opioid and/or benzodiazepine dose and over what time period. Cunningham et al. (2016) [31] and Gilliam et al. (2018) [32] assessed a 3-week intensive interdisciplinary rehabilitation program, whereas Zaman et al. (2018) [33] evaluated an electronic intervention in primary care for US veterans. Due to the differences in deprescribing regimens, settings, and patient populations across the three studies, it is not surprising that there was a large range of successful discontinuation of the opioid, benzodiazepine, or both (21–100%) over a large range of time (3 weeks to 6 months). 

Our study also discovered limited and inconclusive evidence on which drug to deprescribe first among concurrent opioid and benzodiazepine users. While none of the three original research studies identified in our review mention the sequential order for deprescribing the two drugs, two of the identified guidelines recommended that opioids be deprescribed first [35,36], and two guidelines suggested the opposite [34,37]. Most state resources we identified cite the 2016 CDC guideline [64], which suggests tapering opioids first. However, the most recently released 2022 CDC opioid guidelines downgraded the evidence category recommendation for concurrent opioid and benzodiazepine use from Category A “avoid prescribing opioid pain medication and benzodiazepines concurrently” to Category B “caution with concurrent use” and no longer recommend the sequential order for describing these two medications [38]. This recent change in the CDC opioid guidelines emphasizes the importance of (1) risk–benefit evaluations of concurrent use and (2) planning the sequential order and rate of deprescription based on individual patients’ chronic disease conditions and treatment needs [38]. Two guidelines [34,37] recommend or give an example of tapering benzodiazepines first. This may be due to the greater risks involved with benzodiazepine withdrawal than with opioid withdrawal. For example, the Minnesota Department of Human Services states that patients receiving benzodiazepines intermittently with a high daily MME are more likely to successfully deprescribe benzodiazepine first [37]. 

Of the 26 identified guidelines on deprescribing opioids or benzodiazepines, 22 mentioned the risk of concurrent opioid and benzodiazepine use and recommended against concurrent use. In the midst of the US opioid crisis, several agencies have included concurrent opioid and benzodiazepine use as a negative quality indicator to prevent potentially unsafe prescription use. For example, the Centers for Medicare and Medicaid Services (CMS) included concurrent use (defined as ≥30 cumulative days during one year) on the Medicare Part D Display Page to facilitate quality improvement by the plans [65]. It is important to note that, while recommendations are often made to avoid concurrent use from the start, once this therapy has begun, there is a lack of clear guidance and agreement on deprescription other than a suggestion to provide individualized therapy in a safe and effective manner. To improve controlled-substance prescription and reduce patients’ risk of adverse outcomes such as overdose, many states have made it mandatory for prescribers and pharmacists to review a patient’s controlled-substance prescription records through prescription drug monitoring programs. This process provides opportunities to assess other controlled substances that the patient may be using and could impact deprescription initiatives and strategies.

Even though two of the three studies we included successfully deprescribed concurrent opioid and benzodiazepine use over three weeks, those studies’ success can largely be attributed to an intensive rehabilitation program that may not be feasible for most patients and may not be generalizable to patients seen in ambulatory practice. In addition, of the three studies, only Gilliam et al. (2018) reported the patients’ physical and emotional functioning six months after the deprescription. There is still a knowledge gap on the net benefit of deprescribing one or both medications for concurrent users. Our previous research shows that long-term, low-dose, concurrent users were not associated with an increased risk of overdose [66]. Deprescribing strategies are generally driven by safety considerations such as high-dose use (e.g., higher doses associated with falls/fractures and overdose), high-risk populations (e.g., older adults, patients with a history of substance use disorders), likelihood of developing withdrawal symptoms, and patients’ fear of precipitated withdrawal resulting in drug-seeking patterns [36]. Therefore, to minimize negative impacts, clinicians must communicate with patients on deprescribing strategies (e.g., when to continue, pause, or discontinue the taper) and set up clear expectations of deprescribing goals (e.g., full discontinuation vs. maintaining the minimum effective dose). Alternative medication may be considered to manage the original indication, including pain (e.g., gabapentin for patients with neuropathic pain, serotonin and norepinephrine reuptake inhibitors for patients with pain and depression or anxiety) or insomnia (e.g., trazodone for sleep disturbance) and any withdrawal symptoms (e.g., clonidine for blood pressure changes, loperamide for diarrhea). Clinicians must integrate appropriate risk–benefit ratio considerations based on individual patients’ chronic disease conditions and treatment needs with any deprescribing efforts [38]. Another factor influencing deprescribing efforts may be found in the reluctance of physicians to deprescribe or discontinue medications that were initiated or prescribed by other clinicians [67]. To remedy this challenge, there is a need for better communication and care coordination between prescribers. Appropriate coordination of patient care ensures successful and safe concurrent use deprescription, especially among older adults and those with mental health disorders [47].

Two major limitations to this scoping review are the small number of disparate studies/guidelines identified on concurrent use deprescription and the possibility of missing studies/guidelines published through other mechanisms. Although we were unable to draw a systematic recommendation or generate a deprescribing algorithm for concurrent use due to study/guideline disparity and heterogeneity, the three identified peer-reviewed studies consistently reported significant decreases in concurrent use. To mitigate the number of studies/guidelines we might have missed, we included a librarian in our search strategy development, used rigorous methods in our sampling process, and conducted backward searching of the included studies and reviews identified by our searches. Despite these limitations, this scoping review provides valuable insight for deprescribing opioids and benzodiazepines and identifies knowledge gaps for future studies.

## 5. Conclusions

Few deprescribing schedules are available for concurrent opioid and benzodiazepines use, and existing peer-reviewed studies and guidelines are discordant on this important topic. The current, peer-reviewed literature reports significant success for discontinuing one of the two drug classes when including an intensive rehabilitation program and moderate success for discontinuing both drug classes using other interventions. To improve patient safety and quality of care, future studies on deprescribing concurrent use should further clarify optimal strategies, particularly for high-risk populations, such as the elderly and pregnant individuals. Any deprescribing decision must be based on the individual patient’s chronic disease condition and treatment needs.

## Figures and Tables

**Table 1:** Characteristics of unique guidelines and protocols for deprescribing opioids or benzodiazepines.

Source	Target Population	Deprescribing Rate	Organizations Using the Guideline	Elements in the Guidelines/Protocols (Y = Yes; N = No): (a) Risk of Concurrent Use; (b) Criteria for Considering Tapering Opioid/Benzodiazepine Therapy; (c) Shared Decision-Making with Pts; (d) Withdrawal Management; (e) Nonpharmacological Approaches; (f) Alternative Medication Therapies.					
**Opioids (N = 16)**				**(a)**	**(b)**	**(c)**	**(d)**	**(e)**	**(f)**
CDC, 2022 [38]	Pts with CNCP	1. OPI use ≥1 year: reduce dose 10% or less per month. 2. OPI use for shorter durations: reduce initial dose 10% or less per week until ~30%, followed by 10% per week.	*	Y	Y	Y	Y	Y	Y
VA/DoD, 2022 [39]	Pts with CNCP	Gradual and individualized taper: reduce dose 5–20% every 4 weeks or longer.		Y	Y	Y	Y	Y	Y
Minnesota Department of Human Service, 2021 [37]	Pts with CNCP	Individualized taper based on patient tolerance: 1. Slower: reduce dose 5–10% per month to start but may need to reduce rate to less than 5% per month over 2–3 months as tolerated. 2. Faster: reduce initial dose 10% per week or longer; not preferred but used when risk of continuing therapy outweighs risk of rapid taper or when part of treatment program for short time period. 3. May taper BZD first for pts with high daily MME and intermittent BZD.		Y	Y	Y	Y	Y	Y
Oregon Health Authority, 2020 [40]	Pts on OPIs	Individualized: generally, reduce dose 5–10% per month.		Y	Y	Y	Y	Y	Y
Arizona Department of Health Services, 2018 [41]	Pts on LTOT	Individualized taper based on risk assessment: 1. Slowest (over years): reduce dose 2–10% every 4–8 weeks with pauses in taper as needed. 2. Slow (over months to years): reduce dose 5–20% every 4 weeks with pauses in taper as needed. 3. Faster (over weeks): reduce dose 10–20% every week. 4. Rapid (over days): reduce first dose 20–50%, then 10–20% every day.		Y	Y	Y	Y	Y	Y
Cigna, 2019 [42]	Pts on LTOT	Individualized taper based on pt health history, preferences, and risk factors, e.g., reduce dose 5–10% per month.		Y	Y	Y	Y	Y	Y
FDA, 2019 [16]	Pts physically dependent on OPIs	Gradual and individualized taper: 1. No more than 10–25% reduction every 2–4 weeks. 2. More rapid taper: pts on opioids for shorter time periods.	UnitedHealthcare [43]	N	Y	Y	Y	Y	N
HHS, 2019 [44]	Pts on LTOT for chronic pain	1. Common: reduce dose 5–20% every 4 weeks. 2. Gradual: reduce dose 10% per month or slower. 3. Rapid: reduce dose 10% per week up to 30% of the original dose and then 10% per week.	Sunshine Health [45]	Y	Y	Y	Y	Y	Y
Mendoza, 2019 [46]	Pts with CNCP	1. Rapid: reduce dose 20% per week or abrupt discontinuation. 2. Slow: reduce dose 5–20% every 2–4 weeks.		Y	Y	Y	Y	Y	Y
Lumish, 2018 [47]	Pts ≥65 years with CNCP	Reduce dose 5–20% every 4 weeks.		Y	Y	Y	Y	Y	Y
Murphy, 2018 [48]	Pts with CNCP	Reduce dose 5–10% every 2–4 weeks.		Y	Y	Y	Y	Y	Y
Nebraska Department of Health and Human Services, 2017 [35]	Pts on OPIs	1. Long-acting OPIs: reduce dose 5–10% per week. 2. Short-acting OPIs: reduce dose 5–15% per week. 3. After reaching 1/4–1/2 of the initial dose, may slow dose reduction rate for pts cooperative with therapy. 4. Taper opioid first to reduce risk of overdose in pts on both OPI and BZD.		Y	Y	Y	Y	Y	Y
VA PBM, 2016 [49]	Pts on OPIs	1. Slowest: reduce dose 2–10% every 4–8 weeks with pauses in taper as needed (e.g., pts taking high doses of long-acting opioids for many years). 2. Slower: reduce dose 5–20% every 4 weeks. 3. Faster: reduce dose 10–20% per week. 4. Rapid: reduce dose 20–50% of first dose, then 10–20% every day.	United Healthcare [43]	Y	Y	Y	Y	Y	Y
AMDG, 2015 [36]	Pts with CNCP	1. Taper off opioid first and then benzodiazepine with a deprescribing rate based on safety profile. 2. Slow: reduce dose by ≤10% per week for pts with no acute safety concerns from a mental/physical health perspective. 3. Rapid: discontinue over 2–3 weeks if pts having severe adverse outcomes (e.g., overdose or SUD). 4. Immediate: discontinuation if diversion/nonmedical use.	Sunshine Health [45]	Y	Y	Y	Y	Y	Y
Berna, 2015 [50]	Pts with CNCP	Reduce dose 10% every 5–7 days until reaching 30% of the initial dose, then reduce 10% per week.	United Healthcare [43]	N	Y	Y	Y	Y	Y
Oregon Pain Guidance, 2014 [34]	Pts with CNCP	1. Long-acting OPIs: reduce dose 5–10% per week. 2. Short-acting OPIs: reduce dose 5–15% per week. 3. Slow down rate toward the end of taper. Once reaching 25–50% of initial dose, slow to 5% per week. 4. Taper BZD followed by OPI if both drugs involved.	Sunshine Health [45]	Y	Y	Y	Y	Y	Y
**Benzodiazepines (N = 11)**									
Kaiser Permanente, 2022 [51]	Pts on chronic BZD therapy	Individualized taper based on indication: 1. Slow (reduce dose 10% every 2–4 weeks): function not improved or tolerance developed with long-term Rx. 2. Moderate (reduce dose 10% per week): risks greater than benefit or increased risk with comorbidities. 3. Rapid (reduce dose 25% per week): substance abuse, significant risk due to unstable clinical condition or recent overdose or misuse or diversion of medication.		Y	Y	Y	Y	Y	Y
FDA, 2020 [17]	Pts on BZDs	1. Gradual and individualized taper. 2. When experiencing withdrawal symptoms, may pause the taper or raise BZD to previous dose. Once stable, proceed with more gradual taper.		Y	Y	Y	Y	Y	N
Payne, 2019 [52]	Pts on BZDs	1. Reduce dose 10–12.5% every 1–2 weeks over 2–12 months. 2. Reduce dose 10–25% every 2 weeks over 4–8 weeks.		N	Y	Y	Y	Y	N
Presbyterian Healthcare Services, 2019 [53]	Pts ≥18 years on BZD or Z-drug therapy	Taper duration (considering prior use duration) 1. Prior use < 3 months: over 1 week 2. Prior use 3 months–1 year: over 1 month 3. Prior use > 1 year: over 3 months Taper dosage 1. Typical 3-month duration, reduce from 100% to 50% of initial dose during the first 4 weeks, then reduce from 50% to 0% during remaining 2 months. 2. More rapid (25% per week) may be appropriate for pts having increased risk of respiratory depression or misusing/diverting Rx.		Y	Y	Y	Y	Y	Y
Pottie, 2018 [54]	Pts ≥65 years on BZRAs Pts 18–64 years on BZRAs > 4 weeks	Reduce dose 25% every 2 weeks, then 12.5% near end with duration of taper dependent upon patient tolerance, dependence, and potential for withdrawal effects.	Choosing Wisely Canada [55,56] deprescribing.org [57] CalOptima [58]	N	Y	Y	Y	Y	Y
Pruskowski, 2018 [59]	“Older” pts in palliative care on BZD	Reduce over 8–12 weeks total, reduce baseline dose 10–25% every 2–3 weeks based on BZD terminal half-life.		Y	Y	Y	Y	Y	Y
Ogbonna, 2017 [60]	Pts on BZDs daily > 1 month	Reduce initial dose 5–25%, then 5–25% every 1–4 weeks as tolerated; reduce supratherapeutic dose 25–30% as tolerated, followed by 5–10% daily, weekly, or monthly as appropriate. Complex cases may require stabilization at 50% dose reduction for several months prior to resuming taper.	CalOptima [58]	Y	Y	Y	Y	Y	Y
VA PTSD, 2015 [61]	Pts with PTSD	Reduce dose 50% the first 2–4 weeks then maintain dose for 1–2 months. Further reduce dose 25% every 2 weeks.		Y	Y	Y	Y	Y	Y
Oregon Pain Guidance, 2014 [34]	Pts on BZDs	1. Slow: Reduce initial dose 25–50%, then 5–10% per week with follow up; total dose reduction of long-acting drug 5–10% per week in divided doses. When 25–50% of starting dose is reached, slowly taper further to 5% or less per week. 2. Rapid: Pre-medicate for 2 weeks prior with carbamazepine 200 mg every morning and 400 mg every bedtime or valproate 500 mg twice daily. Continue these medications 4 weeks after BZD is discontinued. Discontinue the current BZD and switch to diazepam 2 mg twice daily × 2 days, followed by 2 mg daily × 2 days, then stop. For high doses, may begin with 5 mg twice daily × 2 days and then continue as described. 3. Taper BZD followed by OPI if both drugs involved.	Sunshine Health [45]	Y	Y	Y	Y	Y	Y
Belanger, 2009 [62]	Pts with chronic insomnia	1. Reduce dose by 25% every 1–2 weeks to smallest minimal dose. 2. Switch short-acting BZD to longer-acting BZD. Reduce initial dose 25% by 2nd week, 50% by 4th week, 100% by 10th week.		Y	Y	Y	Y	Y	Y
Woodward, 2003 [63]	Pts ≥ 65 years	1. Short-acting: cease or wean if not needed. 2. Long-acting: reduce dose 10–15% per week. 3. Both 1 and 2: combine with sleep hygiene/psychotherapy.		Y	Y	Y	Y	Y	Y

Abbreviations: BZD: benzodiazepine; BZRA: benzodiazepine receptor agonist; CNCP: chronic noncancer pain; LTOT: long-term opioid therapy; MME: morphine milligram equivalents; OPI: opioid; Pts: patients; Rx: prescription. * United Healthcare [43] and Sunshine Health [45] cite the 2016 version of the CDC guideline.

## Data Availability

The data presented in this study are available in this article in the results section, Table 1 and Tables S2–S4, and Figure 2.

## Supplementary Materials

The following supporting information can be downloaded at https://www.mdpi.com/article/10.3390/jcm12051788/s1: Table S1: PubMed search strategy; Table S2: Opioid and benzodiazepine deprescribing sources identified via state health departments; Table S3: Studies reporting details of deprescribing strategies for opioids or benzodiazepines only by country (n = 36); Table S4. Studies reporting deprescribing strategies for concurrent opioid and benzodiazepine use (n = 3). References [68,69,70,71,72,73,74,75,76,77,78,79,80,81,82,83,84,85,86,87,88,89,90,91,92,93,94,95,96,97,98,99,100,101,102,103] are cited in Supplementary Materials.
