# Supplementary material for: Deprescribing Strategies for Opioids and Benzodiazepines with Emphasis on Concurrent Use: A Scoping Review
Source: J Clin Med. 2023 Feb 23;12(5):1788. doi: 10.3390/jcm12051788 (PMC10002935; doi:10.3390/jcm12051788)
Supplement: Supplementary file 1 [file jcm-12-01788-s001.zip › jcm-2164066-supplementary.pdf]

Supplementary Materials

Table S1. PubMed Search Strategy

Table S2. Opioid and Benzodiazepine Deprescribing Sources Identified via State Health Departments

Table S3. Studies Reporting Details of Deprescribing Strategies for Opioids or Benzodiazepines Only by Country (n=36)

Table S4. Studies Reporting Deprescribing Strategies for Concurrent Opioid and Benzodiazepine Use (n=3)

Table S1. PubMed Search Strategy

Search number	Query	Filters	Results
51	#47 AND #45	English, from 1995 - 2020	2,235
48	#47 AND #45		2,707
47	#44 OR #46		72,339
46	(discontinue[Title] OR discontinued[Title] OR discontinuing[Title] OR lower[Title] OR lowering[Title] OR lowered[Title] OR reduce[Title] OR reducing[Title] OR reduction[Title] OR reduced[Title] OR cease[Title] OR ceasing[Title] OR ceased[Title] OR stop[Title] OR stopping[Title] OR stopped[Title]) AND (medication*[Title] OR medicine[Title] OR therapy[Title] OR treatment[Title] OR dose[title] OR doses[title] OR drug*[Title] OR prescription*[Title])		50,761
45	#43 OR #21		264,903
44	("Deprescriptions"[Mesh]) OR (deprescription[Title/Abstract] OR deprescribe[Title/Abstract] OR deprescribing[Title/Abstract] OR taper[Title/Abstract] OR tapering[Title/Abstract] OR tapered[tiab] OR tapered[Title/Abstract])		21,791
43	#21 OR #22 OR #24 OR #25 OR #27 OR #28 OR #29 OR #30 OR #31 OR #32 OR #33 OR #34 OR #35 OR #36 OR #37 OR #38 OR #39 OR #40 OR #41 OR #42		264,903
42	("Analgesics, Opioid"[Mesh]) OR (opioid[Title/Abstract] OR opioids[Title/Abstract])		109,733
41	("Tapentadol"[Mesh]) OR (tramadol[Title/Abstract] OR Ultram[Title/Abstract] OR Prontofort Zytram[Title/Abstract] OR Takadol[Title/Abstract] OR Theradol[Title/Abstract] OR Tiral OR[Title/Abstract])		5,564
40	("Tapentadol"[Mesh]) OR (tapentadol[Title/Abstract] OR Nucynta[Title/Abstract])		532
39	("Pentazocine"[Mesh]) OR (pentazocine[Title/Abstract] OR Lexir[Title/Abstract] OR Talwin[Title/Abstract] OR Fortral[Title/Abstract])		3,041
38	("Oxymorphone"[Mesh]) OR (oxymorphone[Title/Abstract] OR Numorphan[Title/Abstract] OR Opana[Title/Abstract])		804
37	("Oxycodone"[Mesh]) OR (oxycodone[Title/Abstract] OR Dihydrone[Title/Abstract] OR Oxycone[Title/Abstract] OR Dihydrohydroxycodeinone[Title/Abstract] OR Oxycodeinon[Title/Abstract] OR Eucodal[Title/Abstract] OR Theocodin[Title/Abstract] OR Oxycontin[Title/Abstract] OR Pancodine[Title/Abstract] OR Dinarkon[Title/Abstract] OR Oxiconum OR[Title/Abstract])		4,163
36	("Opium"[Mesh]) OR (opium[Title/Abstract] OR Papaveretum[Title/Abstract] OR Omnopon[Title/Abstract] OR Pantopon[Title/Abstract])		3,793
35	("Morphine"[Mesh]) OR (morphine[Title/Abstract] OR Morphia[Title/Abstract])		57,936
34	("Methadone"[Mesh]) OR (methadone[Title/Abstract] OR Biodone[Title/Abstract] OR Dolophine[Title/Abstract] OR Metadol[Title/Abstract] OR Metasedin[Title/Abstract] OR Symoron[Title/Abstract] OR Methadose[Title/Abstract] OR Methex[Title/Abstract] OR Phenadone[Title/Abstract] OR Physeptone[Title/Abstract] OR Phymet[Title/Abstract] OR Pinadone[Title/Abstract] OR Amidone[Title/Abstract])		16,976
33	("Meperidine"[Mesh]) OR (meperidine[Title/Abstract] OR Pethidine[Title/Abstract] OR Isonipecain[Title/Abstract] OR		7,893

	Dolsin[Title/Abstract] OR Dolosal[Title/Abstract] OR Dolin[Title/Abstract] OR Dolantin[Title/Abstract] OR Dolargan[Title/Abstract] OR Lidol[Title/Abstract] OR Lydol[Title/Abstract] OR Demerol[Title/Abstract] OR Dolcontral[Title/Abstract])	
32	("Levorphanol"[Mesh]) OR (levorphanol[Title/Abstract] OR Levodroman[Title/Abstract] OR Levorphan[Title/Abstract] OR "Levo Dromoran"[Title/Abstract])	843
31	("Methadyl Acetate"[Mesh]) OR ("Levomethadyl acetate"[Title/Abstract] OR "Methadyl Acetate"[Title/Abstract] OR Acetylmethadol[Title/Abstract] OR Alphacetylmethadol[Title/Abstract] OR Amidolacetate[Title/Abstract] OR Dimepheptanol[Title/Abstract] OR Levoacetylmethadol[Title/Abstract] OR Methadol[Title/Abstract] OR Acemethadone[Title/Abstract] OR Levomethadyl[Title/Abstract])	494
30	("Hydromorphone"[Mesh]) OR (Hydromorphone[Title/Abstract] OR Dihydromorphinone[Title/Abstract] OR Hydromorphon[Title/Abstract] OR Palladone[Title/Abstract] OR Laudacon[Title/Abstract] OR Dilaudid[Title/Abstract])	2,201
29	("Hydrocodone"[Mesh]) OR (Hydrocodone[Title/Abstract] OR Hydrocodon[Title/Abstract] OR Dihydrocodeinone[Title/Abstract] OR Dicodid[Title/Abstract] OR Robidone[Title/Abstract] OR Hydrocodeinonebitartrate[Title/Abstract] OR Hydrocon[Title/Abstract] OR Codinovo[Title/Abstract] OR Hycodan[tiab] OR Hycon[Title/Abstract])	1,291
28	("Fentanyl"[Mesh]) OR (Fentanyl[Title/Abstract] OR Phentanyl[Title/Abstract] OR Fentanest[Title/Abstract] OR Duragesic[Title/Abstract] OR Durogesic[Title/Abstract] OR Fentora[Title/Abstract] OR Sublimaze[Title/Abstract])	24,954
27	("dihydrocodeine" [Supplementary Concept]) OR (dihydrocodeine[Title/Abstract] OR Rikodeine[Title/Abstract] OR [Title/Abstract] OR Tiamon[Title/Abstract] OR Tosidrin[Title/Abstract] OR Contugesic[Title/Abstract] OR Paracodin[Title/Abstract] OR Paracodina[Title/Abstract])	390
25	("Codeine"[Mesh]) OR (Codeine[Title/Abstract] OR Ardinex[Title/Abstract] OR Isocodeine[Title/Abstract])	9,418
24	("Butorphanol"[Mesh]) OR (Butorphanol[Title/Abstract] OR Beforal[Title/Abstract] OR Moradol[Title/Abstract] OR Stadol[Title/Abstract] OR Torbugesic[Title/Abstract] OR "Apo-Butorphanol"[Title/Abstract] OR Dolorex[Title/Abstract])	1,659
22	"Buprenorphine"[Mesh] OR Buprenorphine[tiab] OR Buprenex[tiab] OR Prefin[tiab] OR Subutex[tiab] OR Buprex[tiab] OR Temgesic[tiab]	7,832
21	#1 OR #2 OR #3 OR #4 OR #5 OR #6 OR #7 OR #8 OR #9 OR #10 OR #11 OR #12 OR #13 OR #14 OR #15 OR #16 OR #17 OR #18 OR #19 OR #20	91,621
20	"Prazepam"[Mesh] OR Prazepam[tiab] OR Lysanxia[tiab] OR Reapam[tiab] OR Centrax[tiab] OR Demetrin[tiab]	228
19	"Benzodiazepines"[Mesh] OR benzodiazepine*[tiab]	80,241
18	"Eszopiclone"[Mesh] OR Eszopiclone[tiab] OR Lunesta[tiab] OR Estorra[tiab]	281
17	"zopiclone" [Supplementary Concept] OR Zopiclone[tiab] OR Zopitan[tiab] OR Zorclone[tiab] OR Imovane[tiab] OR Ximovan[tiab] OR Zimovane[tiab] OR Limovan[tiab] OR Rhovane[tiab]	1,091
16	"zaleplon" [Supplementary Concept] OR Zaleplon[tiab] OR Zelepion[tiab] OR Starnoc[tiab] OR sonata[tiab]	664
15	"Zolpidem"[Mesh] OR Zolpidem[tiab] OR Zolirin[tiab] OR Zolpinox[tiab] OR Zolpimist[tiab] OR Ambien[tiab] OR Amsic[tiab] OR Bikalm[tiab] OR	2,528

	Stilnoct[tiab] OR Stilnox[tiab] OR Dalparan[tiab] OR Zodormdura[tiab] OR Zoldem[tiab]	
14	"Triazolam"[Mesh] OR Triazolam[tiab] OR Halcion[tiab] OR Trilam[tiab] "Temazepam"[Mesh] OR Temazepam[tiab] OR Hydroxydiazepam[tiab] OR Oxydiazepam[tiab] OR Methyloxazepam[tiab] OR Planum[tiab] OR Remestan[tiab] OR Restoril[tiab] OR Levanxol[tiab]	2,255
13	"quazepam" [Supplementary Concept] OR Quazepam[tiab] OR Quiedorm[tiab] OR Doral[tiab]	184
12	"Oxazepam"[Mesh] OR Oxazepam[tiab] OR Serax[tiab] OR Tazepam[tiab] OR Adumbran[tiab]	1,962
11	"halazepam" [Supplementary Concept] OR halazepam[tiab]	60
10	"Nitrazepam"[Mesh] OR Nitrazepam[tiab] OR Nitrodiazepam[tiab] OR "Dormo-Puren"[tiab] OR Eatan[tiab] OR Imadorm[tiab] OR Imeson[tiab] OR Mogadon[tiab] OR Nitrazadon[tiab] OR Nitrazep[tiab] OR Novanox[tiab] OR Radedorm[tiab] OR Remnos[tiab] OR Serenade[tiab] OR Somnite[tiab] OR Alodorm[tiab] OR Dormalon[tiab]	1,354
9	"Lorazepam"[Mesh] OR lorazepam[tiab] OR Lorazapam[tiab] OR Ativan[tiab] OR "Orfidal Wyeth"[tiab] OR Temesta[tiab] OR Tolid[tiab] OR Donix[tiab] OR Duralozam[tiab] OR Durazolam[tiab] OR [tiab] OR Idalprem[tiab] OR Laubeel[tiab]	2,196
8	"Flurazepam"[Mesh] OR Flurazepam[tiab] OR Dalmane[tiab] OR Dalmadorm[tiab] OR Dormodor[tiab] OR "Apo Flurazepam"[tiab]	1,184
7	"Estazolam"[Mesh] OR Estazolam[tiab] OR Tasedan[tiab] OR ProSom[tiab] OR Nuctalon[tiab]	251
6	"Diazepam"[Mesh] OR Diazepam [tiab] OR Diazemuls[tiab] OR Faustan[tiab] OR Valium[tiab] OR Seduxen[tiab] OR Sibazon[tiab] OR Stesolid[tiab] OR Apaurin[tiab] OR Relanium[tiab]	25,859
5	"Clorazepate Dipotassium"[Mesh] OR Clorazepate[tiab] OR "clorazepate dipotassium"[tiab] OR Chlorazepate[tiab] OR Clorazepate[tiab] OR Tranxene[tiab] OR Tranxilium[tiab] OR "Clorazepic Acid"[tiab]	543
4	"Clonazepam"[Mesh] OR Clonazepam[tiab] OR Antelepsin[tiab] OR Rivotril[tiab]	4,570
3	"Chlordiazepoxide"[Mesh] OR Chlordiazepoxide[tiab] OR Methaminodiazepoxide[tiab] OR Librium[tiab] OR Chlozepid[tiab] OR Elenium[tiab]	4,819
2	"Bromazepam"[Mesh] OR Bromazepam[tiab] OR BromaLich[tiab] OR Bromazanil[tiab] OR Durazanil[tiab] OR Lexotan[tiab] OR Lexomil[tiab] OR Lexatin[tiab] OR Lexotanil[tiab] OR Anxyrex[tiab]	593
1	"Alprazolam"[Mesh] OR alprazolam[tiab] OR Alprazolan[tiab] OR Trankimazin[tiab] OR Xanax[tiab] OR Alprox[tiab] OR Esparon[tiab] OR Cassadan[tiab] OR Kalma[tiab] OR "Novo-Alprazol"[tiab] OR "Novo Alprazol"[tiab] OR "Nu-Alpraz"[tiab] OR "Nu Alpraz"[tiab] OR Ralozam[tiab] OR Tafil[tiab] OR "Apo-Alpraz"[tiab] OR "Apo Alpraz"[tiab]	2,724

Complete search strategies are available upon request.

Table S2. Opioid and Benzodiazepine Deprescribing Sources Identified Using State Health Department Websites

State	URL for OPI deprescribing recommendations	OPI Source	URL for BZD deprescribing recommendations	BZD Source
Alabama	https://www.alabamapublichealth.gov/pdmp/educational-materials.html	CDC	NR	NR
Alaska	https://dhss.alaska.gov/dph/Director/Pages/opioids/prescribers.aspx	CDC; HHS	NR	NR
Arizona	https://www.azdhs.gov/audiences/clinicians/index.php#clinical-guidelines-and-references-rx-guidelines https://www.azdhs.gov/documents/audiences/clinicians/clinical-guidelines-recommendations/prescribing-guidelines/az-opioid-prescribing-guidelines.pdf	CDC; VA/DoD	NR	NR
Arkansas	NR	NR	NR	NR
California	https://www.cdph.ca.gov/Programs/CCDPHP/DCDIC/SACB/Pages/Resources.aspx	CDC	NR	NR
Colorado	NR	NR	NR	NR
Connecticut	NR	NR	NR	NR
Delaware	NR	NR	NR	NR
Florida	NR	NR	NR	NR
Georgia	https://dph.georgia.gov/stopopioidaddiction/medical-providers-and-pharmacists	CDC	NR	NR
Hawaii	NR	NR	NR	NR
Idaho	https://healthandwelfare.idaho.gov/providers/opioid-prescribing/opioid-prescribing-providers<br><br>https://healthandwelfare.idaho.gov/providers/opioid-prescribing/provider-resources	CDC; HHS	NR	NR
Illinois	https://dph.illinois.gov/topics-services/opioids/prescription-opioids-and-heroin.html	CDC	NR	NR
Indiana	https://www.in.gov/health/overdose-prevention/resources-for-medical-professionals/opioid-prescribing-guidelines/	CDC	NR	NR
Iowa	https://idph.iowa.gov/Bureau-of-Substance-Abuse/Prevention-Related-Programs/Current-Grants/Strategic-Initiatives-to-Prevent-Drug-Overdoses-Grant/Academic-Detailing-Resources	CDC	NR	NR
Kansas	NR	NR	NR	NR
Kentucky	NR	NR	NR	NR

State	URL for OPI deprescribing recommendations	OPI Source	URL for BZD deprescribing recommendations	BZD Source
Louisiana	https://ldh.la.gov/page/2755	CDC	NR	NR
Maine	NR	NR	NR	NR
Maryland	https://health.maryland.gov/bha/Documents/CDC%20Info.pdf#search=opioid%20prescribing<br><br>https://health.maryland.gov/pha/Documents/CDC%201%20_guidelines_factsheet.pdf#search=opioid%20prescribing<br><br>https://health.maryland.gov/pdmp/Pages/-Clinical-Resources.aspx	CDC; HHS	NR	NR
Massachusetts	NR	NR	NR	NR
Michigan	https://www.michigan.gov/documents/mdhhs/CDC_Guidelines_Prescribing_Opioids_618282_7.pdf<br><br>https://www.michigan.gov/documents/mdhhs/Checklist_for_prescribing_opioids_618296_7.pdf	CDC	NR	NR
Minnesota	https://mn.gov/dhs/opip/provider-education/<br><br>https://edocs.dhs.state.mn.us/lfserver/Public/DHS-7757C-ENG<br><br>https://mn.gov/dhs/opip/opioid-guidelines/tapering-opioids/	HHS; AMDG; Berna 2015; CDC; VA/DoD	NR	NR
Mississippi	NR	NR	NR	NR
Missouri	https://health.mo.gov/safety/bndd/pdf/opiate-guidelines.pdf	CDC	NR	NR
Montana	NR	NR	NR	NR
Nebraska	https://dhhs.ne.gov/Guidance%20Docs/Pain%20Management%20Pain%20Guidance%20Document.pdf#search=opioid%20prescribing	CDC; OPG	https://dhhs.ne.gov/Guidance%20Docs/Pain%20Management%20Pain%20Guidance%20Document.pdf#search=opioid%20prescribing	OPG
Nevada	https://dpbh.nv.gov/Resources/opioids/Prescription_Drug_Abuse_Prevention/	CDC	NR	NR
New Hampshire	NR	NR	NR	NR
New Jersey	NR	NR	NR	NR
New Mexico	https://www.nmhealth.org/publication/view/help/2244/<br><br>https://www.nmhealth.org/about/erd/ibeb/pos/ospr/	CDC	https://www.nmhealth.org/publication/view/guide/6327/	Kaiser Permanente; Pottie 2018
New York	https://www.health.ny.gov/professionals/narcotic/opioid_treatment_guidelines/	CDC	NR	NR

State	URL for OPI deprescribing recommendations	OPI Source	URL for BZD deprescribing recommendations	BZD Source
North Carolina	https://www.ncdhhs.gov/media/1755/download	CDC	NR	NR
North Dakota	NR	NR	NR	NR
Ohio	https://mha.ohio.gov/static/Portals/0/assets/ResearchersAndMedia/Combating%20Opiate%20Abuse/OPG/Acute-pain-infographic.pdf?ver=2018-11-08-125608-507<br><br>https://mha.ohio.gov/static/Portals/0/assets/ResearchersAndMedia/Combating%20Opiate%20Abuse/OPG/Guidelines-Chronic-Pain.pdf?ver=2018-11-08-133715-657	CDC; HHS	NR	NR
Oklahoma	https://oklahoma.gov/content/dam/ok/en/health/health2/documents/pocket-guide-tapering.pdf	CDC	NR	NR
Oregon	https://www.oregon.gov/oha/PH/PREVENTIONWELLNESS/SUBSTANCEUSE/OPIOIDS/Pages/task-force.aspx<br><br>https://www.oregon.gov/omb/Topics-of-Interest/Documents/Oregon-Opioid-Tapering-Guidelines.pdf	CDC; HHS	NR	NR
Pennsylvania	https://www.health.pa.gov/topics/programs/Patient-Advocacy/Pages/Healthcare.aspx	CDC; HHS	https://www.health.pa.gov/topics/programs/Patient-Advocacy/Pages/Healthcare.aspx<br><br>https://alosahealth.org/wp-content/uploads/2021/06/Benzo-taper-tool-2021.pdf	Pottie 2018
Rhode Island	https://health.ri.gov/publications/quickreferenceguides/QuickReferenceToRIUpdatedPainManagementRegulations.pdf	CDC	NR	NR
South Carolina	https://scdhec.gov/opioid-epidemic	CDC	NR	NR
South Dakota	https://doh.sd.gov/documents/news/SDOpioidAbuseStrategicPlan.pdf	CDC	NR	NR
Tennessee	https://www.tn.gov/content/dam/tn/health/healthprofboards/pain-management-clinic/ChronicPainGuidelines.pdf	HHS	NR	NR
Texas	https://www.dshs.texas.gov/features/substance-use-action-plan/DSHS-SubstanceUse-ActionPlan.pdf	HHS	NR	NR
Utah	https://opidemic.org/providers/	CDC; AMDG	NR	NR

State	URL for OPI deprescribing recommendations	OPI Source	URL for BZD deprescribing recommendations	BZD Source
Vermont	https://www.healthvermont.gov/sites/default/files/documents/pdf/REG_opioids-prescribing-for-pain.pdf	HHS	NR	NR
Virginia	https://www.vdh.virginia.gov/home/the-opioid-addiction-crisis-is-a-public-health-emergency-in-virginia/	CDC	NR	NR
Washington	https://doh.wa.gov/sites/default/files/legacy/Documents/2300/2017//AMDG-Guidelines.pdf<br><br>https://doh.wa.gov/sites/default/files/legacy/Documents/2600/2017//PrescribingGLcomparisonCDC-AMDG.pdf	CDC; HHS	NR	NR
West Virginia	https://dhhr.wv.gov/News/Pages/DHHR-Announces-Implementation-of-New-Opioid-Prescribing-Requirements.aspx	CDC	NR	NR
Wisconsin	https://www.dhs.wisconsin.gov/opioids/professionals.htm	CDC	NR	NR
Wyoming	https://health.wyo.gov/publichealth/prevention/substanceabuseandsuicide/publications-and-reports/	CDC	NR	NR

Abbreviations: **BZD**: benzodiazepine; **NR**: not reported; **OPI**: opioid

Table S3. Studies Reporting Details of Deprescribing Strategies for Opioids or Benzodiazepines Only by Country (n=36)*

Drug to deprescribe	Country	Studies
Opioid	USA (n=3)	Darnall (2018); Goodman (2018); Sullivan (2017)
	Europe (n=2)	Kurita (2018); Wang (2011)
Benzodiazepine	USA (n=8)	Fung (2019); Gorenstein (2005); Hadley (2012); Otto (1993); Rickels (2000); Rosenbaum (1997); Roy-Byrne (2003); Rynn (2003)
	Canada (n=10)	Baillargeon (2003); Belanger (2005); Belleville (2007); Dellechiaie (1995); Gosselin (2006); Morin (1995); Morin (2004); Morin (2005); O'Connor (2008); Tannenbaum (2014)
	Europe (n=9)	Baandrup (2016); Curran (2003); Mercier-Guyon (2004); OudeVoshaar (2003); Rubio (2011); Vicens (2006); Vicens (2014); Vorma (2002); Zitman (2001)
	Other (n=4)	Cardinali (2002); Kitajima (2012); Nakao (2006); Yang (2015)

*Studies are alphabetically ordered

Table S4. Studies Reporting Deprescribing Strategies for Concurrent Opioid and Benzodiazepine Use (n=3)

Study (year, country)	Study design	Study sample/cohort	OPI-BZD deprescribing protocol	Outcomes evaluated	Other details
Cunningham (2016, USA)	Prospective pre/post without control	55 CNCP pts with fibromyalgia receiving a 3-wk (8hr/weekday) interdisciplinary rehabilitation program at the Mayo Clinic Pain Rehabilitation Center taking a daily OPI with 61% on daily OPI for a mean duration of 4.6 (SD=5.8) years (BZDs use not detailed)	**OPIs:** individualized schedule based on current formulation and dose at program admission. Reduce daily OPI dose during days of program participation and avoid weekend reductions (pts away from the program). Reduce initial OPI dose by 10%–20% each week day during the first half to two-thirds of the deprescribing period, and then by 2.5%–10% each week day during the remaining deprescribing period.<br>• Initial reductions could be >10%–20%, until decreased by 50%–80% of initial daily dose.<br>• Slower tapers: for those with long-term opioid use (>2 years of daily use).<br>**BZDs**: taper schedule individualized during the 3-wk program, then at discharge clinical note recommended the primary care provider continue the current dose for the next 2-4 weeks and then continue to taper the patient by approximately 25% every 2-4 weeks until completed (Julie Cunningham, PharmD, RPh, e-mail communication, September 22, 2022).	• 51 out of 55 (92.7%) discontinued OPIs within 3 wks<br>• Mean tapering durations between pts with <100 vs. >200 MME/day: 10 vs. 18 days (p<0.001).<br>• Significant improvements: numeric pain scores, depression catastrophizing, health perception, interference with life, and perceived life control at program completion	The 3-wk interdisciplinary rehabilitation program also included:<br>• 8 hours of participation at clinic on week days<br>• Cognitive behavioral therapy<br>• Occupational therapy<br>• Biofeedback and relaxation training<br>• Group therapy<br>• Clonidine based on specific withdrawal symptoms<br>• Meetings with interdisciplinary treatment team twice weekly to discuss treatment progress<br>• Withdrawal assessment
Gilliam (2018, USA)	Prospective pre-post without control	344 eligible, CNCP pts receiving a 3-wk (8hr/weekday) interdisciplinary rehabilitation program at the Mayo Clinic Pain Rehabilitation Center; the mean duration of opioid use in the opioid group was 5.8 (SD=4.9) years; 101 of 285 who completed the program were taking BZDs at pre-treatment)	Same as Cunningham (2016, USA)	• **OPIs:** 142 of 165 (86.1%) completed treatment and discontinued within 3 wks<br>• **BZDs**: 23 of 43 (53.4%) discontinued--deprescribing duration not described<br>• **OPI-BZD use**: 20 of 58 (34.5%) co-prescribed BZDs discontinued both drug classes--deprescribing duration not described; 58 of 58 (100%) discontinued opioids.	The 3-wk interdisciplinary rehabilitation program also included:<br>• 8 hours of participation at clinic on week days<br>• Physical and occupational therapy<br>• Individual and group-based CBT sessions<br>• Weekly individual meetings with the multidisciplinary team to discuss treatment progress<br>• Daily withdrawal assessment during taper and several days afterwards
Zaman et al. (2018, USA)	Prospective pre-post	145 veterans with pain and ≥1 overlapping day of OPI-BZD.	**OPIs**: individualized schedule or to reduce to OPI <100 MME/day.	At 6-month follow-up (% change from baseline):	Provider centered electronic interventions provided:

	without control	Exclusion: Pts prescribed with OPIs <90 days in the past 120 days, sublingual buprenorphine, methadone for OUD, a one-time BZD prior to a procedure, or in oncology or palliative care	• Gradual (most patients): reduce dose by 10%–25% every 1–4 wks.<br>• Rapid (medically dangerous situations): reduce dose every 1–7 days.<br>**BZDs**: Individualized schedule<br>• Switch to a longer-acting BZD if no high-dose OPI use<br>• Reduce dose by 50%/wk for the first 2–4 wks, then maintain dose for 1–2 months, then reduce dose by 25% every 2 wks<br>• Avoid prolonged tapers over 6 months	• ↓ No. OPI-BZD pts (33%, p=0.003)<br>• ↓ No. of pts ⩾ 100 MME/day (30%, p<0.001), and ↓ mean MME/day (22%, p<0.001)<br>• ↓ mean DEDD (17%, p<0.001)<br>• ↑ No. pts receiving OEND (94%, p<0.001)<br>• No significant changes in the following outcomes: annual UDS, PDMP review, or signed OIC	• Standard education on the risks of OPI-BZD uses<br>• Individualized safety recommendations (overdose education, naloxone distribution, annual UDS, annual PDMP review, and signed OIC)<br>• Encrypted e-mail to clinicians with a list of pts with OPI-BZD use, taper guide, alternative therapies, and withdrawal symptom management strategies.<br>• EMR note and email asked clinicians to "consider benzodiazepine and/or opioid taper (to <100 MME/day or off entirely if still prescribed benzodiazepine)".

Abbreviations: **BZD**: benzodiazepine; **CNCP:** chronic non-cancer pain; **DEDD:** diazepam equivalent daily dose; **MME**: morphine milligram equivalents; **No.**: number of; **NR**: not reported; **OEND**: opioid overdose education and naloxone distribution; **OIC**: opioid informed consent; **OPI**: opioid; **PDMP**: prescription drug monitoring program; **Pts**: patients; **SD**: standard deviation; **UDS**: urine drug screening; **wk**: week

Supplementary Reference

Baandrup, L., Glenthoj, B. Y., & Jennum, P. J. (2016). Objective and subjective sleep quality: Melatonin versus placebo add-on treatment in patients with schizophrenia or bipolar disorder withdrawing from long-term benzodiazepine use. *Psychiatry Res.*, *240*, 163-169. https://doi.org/10.1016/j.psychres.2016.04.031

Baillargeon, L., Landreville, P., Verreault, R., Beauchemin, J. P., Gregoire, J. P., & Morin, C. M. (2003). Discontinuation of benzodiazepines among older insomniac adults treated with cognitive-behavioural therapy combined with gradual tapering: a randomized trial. *Can. Med. Assoc. J.*, *169*(10), 1015-1020. <Go to ISI>://WOS:000186592000008

Belanger, L., Morin, C. M., Bastien, C., & Ladouceur, R. (2005). Self-efficacy and compliance with benzodiazepine taper in older adults with chronic insomnia. *Health Psychol.*, *24*(3), 281-287. https://doi.org/10.1037/0278-6133.24.3.281

Belleville, G., Guay, C., Guay, B., & Morin, C. M. (2007). Hypnotic taper with or without self-help treatment of insomnia: a randomized clinical trial. *Journal of consulting and clinical psychology*, *75*(2), 325-335. https://doi.org/10.1037/0022-006x.75.2.325

Cardinali, D. P., Gvozdenovich, E., Kaplan, M. R., Fainstein, I., Shifis, H. A., Pérez Lloret, S., Albornoz, L., & Negri, A. (2002). A double blind-placebo controlled study on melatonin efficacy to reduce anxiolytic benzodiazepine use in the elderly. *Neuro endocrinology letters*, *23*(1), 55-60.

Cunningham, J. L., Evans, M. M., King, S. M., Gehin, J. M., & Loukianova, L. L. (2016). Opioid Tapering in Fibromyalgia Patients: Experience from an Interdisciplinary Pain Rehabilitation Program. *Pain medicine (Malden, Mass.)*, *17*(9), 1676-1685. https://doi.org/10.1093/pm/pnv079

Curran, H. V., Collins, R., Fletcher, S., Kee, S. C., Woods, B., & Iliffe, S. (2003). Older adults and withdrawal from benzodiazepine hypnotics in general practice: effects on cognitive function, sleep, mood and quality of life. *Psychological medicine*, *33*(7), 1223-1237. https://doi.org/10.1017/s0033291703008213

Darnall, B. D., Ziadni, M. S., Stieg, R. L., Mackey, I. G., Kao, M. C., & Flood, P. (2018). Patient-Centered Prescription Opioid Tapering in Community Outpatients With Chronic Pain. *JAMA internal medicine*, *178*(5), 707-708. https://doi.org/10.1001/jamainternmed.2017.8709

Dellechiaie, R., Pancheri, P., Casacchia, M., Stratta, P., Kotzalidis, G. D., & Zibellini, M. (1995). ASSESSMENT OF THE EFFICACY OF BUSPIRONE IN PATIENTS AFFECTED BY GENERALIZED ANXIETY DISORDER, SHIFTING TO BUSPIRONE FROM PRIOR TREATMENT WITH LORAZEPAM - A PLACEBO-CONTROLLED, DOUBLE-BLIND-STUDY. *J. Clin. Psychopharmacol.*, *15*(1), 12-19. https://doi.org/10.1097/00004714-199502000-00003

Fung, C. H., Martin, J. L., Alessi, C., Dzierzewski, J. M., Cook, I. A., Moore, A., Grinberg, A., Zeidler, M., & Kierlin, L. (2019). Hypnotic Discontinuation Using a Blinded (Masked) Tapering Approach: A Case Series. *Frontiers in psychiatry*, *10*, 717. https://doi.org/10.3389/fpsyt.2019.00717

Gilliam, W. P., Craner, J. R., Cunningham, J. L., Evans, M. M., Luedtke, C. A., Morrison, E. J., Sperry, J. A., & Loukianova, L. L. (2018). Longitudinal Treatment Outcomes for an Interdisciplinary Pain Rehabilitation Program: Comparisons of Subjective and Objective Outcomes on the Basis of Opioid Use Status. *The journal of pain : official journal of the American Pain Society*, *19*(6), 678-689. https://doi.org/10.1016/j.jpain.2018.02.010

Goodman, M. W., Guck, T. P., & Teply, R. M. (2018). Dialing back opioids for chronic pain one conversation at a time. *The Journal of family practice*, *67*(12), 753-757.

Gorenstein, E. E., Kleber, M. S., Mohlman, J., Dejesus, M., Gorman, J. M., & Papp, L. A. (2005). Cognitive-behavioral therapy for management of anxiety and medication taper in older adults. *The American journal of geriatric psychiatry : official journal of the American Association for Geriatric Psychiatry*, *13*(10), 901-909. https://doi.org/10.1176/appi.ajgp.13.10.901

Gosselin, P., Ladouceur, R., Morin, C. M., Dugas, M. J., & Baillargeon, L. (2006). Benzodiazepine discontinuation among adults with GAD: A randomized trial of cognitive-behavioral therapy. *Journal of consulting and clinical psychology*, *74*(5), 908-919. https://doi.org/10.1037/0022-006x.74.5.908

Hadley, S. J., Mandel, F. S., & Schweizer, E. (2012). Switching from long-term benzodiazepine therapy to pregabalin in patients with generalized anxiety disorder: a double-blind, placebo-controlled

trial. *Journal of psychopharmacology (Oxford, England)*, *26*(4), 461-470. https://doi.org/10.1177/0269881111405360

Kitajima, R., Miyamoto, S., Tenjin, T., Ojima, K., Ogino, S., Miyake, N., Fujiwara, K., Funamoto, Y., Arai, J., Tsukahara, S., & et al. (2012). Effects of tapering of long-term benzodiazepines on cognitive function in patients with schizophrenia receiving a second-generation antipsychotic. *Progress in neuro-psychopharmacology & biological psychiatry*, *36*(2), 300-306. https://doi.org/10.1016/j.pnpbp.2011.11.008

Kurita, G. P., Hojsted, J., & Sjogren, P. (2018). Tapering off long-term opioid therapy in chronic non-cancer pain patients: A randomized clinical trial. *Eur. J. Pain*, *22*(8), 1528-1543. https://doi.org/10.1002/ejp.1241

Mercier-Guyon, C., Chabannes, J. P., & Saviuc, P. (2004). The role of captodiamine in the withdrawal from long-term benzodiazepine treatment. *Current medical research and opinion*, *20*(9), 1347-1355. https://doi.org/10.1185/030079904125004457

Morin, C. M., Bastien, C., Guay, B., Radouco-Thomas, M., Leblanc, J., & Vallieres, A. (2004). Randomized clinical trial of supervised tapering and cognitive behavior therapy to facilitate benzodiazepine discontinuation in older adults with chronic insomnia. *Am. J. Psychiat.*, *161*(2), 332-342. https://doi.org/10.1176/appi.ajp.161.2.332

Morin, C. M., Belanger, L., Bastien, C., & Vallieres, A. (2005). Long-term outcome after discontinuation of benzodiazepines for insomnia: a survival analysis of relapse. *Behav. Res. Ther.*, *43*(1), 1-14. https://doi.org/10.1016/j.brat.2003.12.002

Morin, C. M., Colecchi, C. A., Ling, W. D., & Sood, R. K. (1995). Cognitive behavior therapy to facilitate benzodiazepine discontinuation among hypnotic-dependent patients with insomnia. *Behavior Therapy*, *26*(4), 733-745. https://doi.org/10.1016/S0005-7894(05)80042-5

Nakao, M., Takeuchi, T., Nomura, K., Teramoto, T., & Yano, E. (2006). Clinical application of paroxetine for tapering benzodiazepine use in non-major-depressive outpatients visiting an internal medicine clinic. *Psychiatry Clin Neurosci*, *60*(5), 605-610. https://doi.org/10.1111/j.1440-1819.2006.01565.x

O'Connor, K., Marchand, A., Brousseau, L., Aardema, F., Mainguy, N., Landry, P., Savard, P., Léveillé, C., Lafrance, V., Boivin, S., & et al. (2008). Cognitive-behavioural, pharmacological and psychosocial predictors of outcome during tapered discontinuation of benzodiazepine. *Clinical psychology & psychotherapy*, *15*(1), 1-14. https://doi.org/10.1002/cpp.556

Oude Voshaar, R. C., Gorgels, W., Mol, A. J. J., Van Balkom, A., Van de Lisdonk, E. H., & Breteler, M. H. M. (2003). Tapering off long-term benzodiazepine use with or without group cognitive-behavioural therapy: three-condition, randomised controlled trial. *British journal of psychiatry*, *182*(JUNE), 498-504. https://www.cochranelibrary.com/central/doi/10.1002/central/CN-00476845/full

Rickels, K., DeMartinis, N., Garcia-Espana, F., Greenblatt, D. J., Mandos, L. A., & Rynn, M. (2000). Imipramine and buspirone in treatment of patients with generalized anxiety disorder who are discontinuing long-term benzodiazepine therapy. *American Journal of Psychiatry*, *157*(12), 1973-1979. https://doi.org/10.1176/appi.ajp.157.12.1973

Rosenbaum, J. F., Moroz, G., & Bowden, C. L. (1997). Clonazepam in the treatment of panic disorder with or without agoraphobia: a dose-response study of efficacy, safety, and discontinuance. Clonazepam Panic Disorder Dose-Response Study Group. *Journal of Clinical Psychopharmacology*, *17*(5), 390-400. https://doi.org/10.1097/00004714-199710000-00008

Roy-Byrne, P., Russo, J., Pollack, M., Stewart, R., Bystrisky, A., Bell, J., Rosenbaum, J., Corrigan, M. H., Stolk, J., Rush, A. J., & Ballenger, J. (2003). Personality and symptom sensitivity predictors of alprazolam withdrawal in panic disorder. *Psychological medicine*, *33*(3), 511-518. https://doi.org/10.1017/s0033291703007402

Rubio, G., Bobes, J., Cervera, G., Teran, A., Perez, M., Lopez-Gomez, V., & Rejas, J. (2011). Effects of Pregabalin on Subjective Sleep Disturbance Symptoms during Withdrawal from Long-Term Benzodiazepine Use. *Eur. Addict. Res.*, *17*(5), 262-270. https://doi.org/10.1159/000324850

Rynn, M., Garcia-Espana, F., Greenblatt, D. J., Mandos, L. A., Schweizer, E., & Rickels, K. (2003). Imipramine and buspirone in patients with panic disorder who are discontinuing long-term

benzodiazepine therapy. *J. Clin. Psychopharmacol.*, *23*(5), 505-508. https://doi.org/10.1097/01.jcp.0000088907.24613.3f

Schweizer, E., Case, W. G., Garciaespana, F., Greenblatt, D. J., & Rickels, K. (1995). PROGESTERONE COADMINISTRATION IN PATIENTS DISCONTINUING LONG-TERM BENZODIAZEPINE THERAPY - EFFECTS ON WITHDRAWAL SEVERITY AND TAPER OUTCOME. *Psychopharmacology*, *117*(4), 424-429. https://doi.org/10.1007/bf02246214

Sullivan, M. D., Turner, J. A., DiLodovico, C., D'Appollonio, A., Stephens, K., & Chan, Y. F. (2017). Prescription Opioid Taper Support for Outpatients With Chronic Pain: A Randomized Controlled Trial. *J Pain*, *18*(3), 308-318. https://doi.org/10.1016/j.jpain.2016.11.003

Tannenbaum, C., Martin, P., Tamblyn, R., Benedetti, A., & Ahmed, S. (2014). Reduction of inappropriate benzodiazepine prescriptions among older adults through direct patient education: the EMPOWER cluster randomized trial. *JAMA internal medicine*, *174*(6), 890-898. https://doi.org/10.1001/jamainternmed.2014.949

Vicens, C., Bejarano, F., Sempere, E., Mateu, C., Fiol, F., Socias, I., Aragonès, E., Palop, V., Beltran, J. L., Piñol, J. L., Lera, G., Folch, S., Mengual, M., Basora, J., Esteva, M., Llobera, J., Roca, M., Gili, M., & Leiva, A. (2014). Comparative efficacy of two interventions to discontinue long-term benzodiazepine use: cluster randomised controlled trial in primary care. *The British journal of psychiatry : the journal of mental science*, *204*(6), 471-479. https://doi.org/10.1192/bjp.bp.113.134650

Vicens, C., Fiol, F., Llobera, J., Campoamor, F., Mateu, C., Alegret, S., & Socias, I. (2006). Withdrawal from long-term benzodiazepine use: randomised trial in family practice. *Br. J. Gen. Pract.*, *56*(533), 958-963.

Vorma, H., Naukkarinen, H., Sarna, S., & Kuoppasalmi, K. (2002). Treatment of out-patients with complicated benzodiazepine dependence: comparison of two approaches. *Addiction (Abingdon, England)*, *97*(7), 851-859. https://doi.org/10.1046/j.1360-0443.2002.00129.x

Wang, H., Akbar, M., Weinsheimer, N., Gantz, S., & Schiltenwolf, M. (2011). Longitudinal observation of changes in pain sensitivity during opioid tapering in patients with chronic low-back pain. *Pain medicine (Malden, Mass.)*, *12*(12), 1720-1726. https://doi.org/10.1111/j.1526-4637.2011.01276.x

Yang, C. M., Tseng, C. H., Lai, Y. S., & Hsu, S. C. (2015). Self-efficacy enhancement can facilitate hypnotic tapering in patients with primary insomnia. *Sleep and Biological Rhythms*, *13*(3), 242-251. https://doi.org/10.1111/sbr.12111

Zaman, T., Rife, T. L., Batki, S. L., & Pennington, D. L. (2018). An electronic intervention to improve safety for pain patients co-prescribed chronic opioids and benzodiazepines. *Substance abuse*, *39*(4), 441-448. https://doi.org/10.1080/08897077.2018.1455163

Zitman, F. G., & Couvee, J. E. (2001). Chronic benzodiazepine use in general practice patients with depression: an evaluation of controlled treatment and taper-off - Report on behalf of the Dutch Chronic Benzodiazepine Working Group. *Br. J. Psychiatry*, *178*, 317-324. https://doi.org/10.1192/bjp.178.4.317
